# The Prognostic Value of Cancer Stem Cell Markers (CSCs) Expression—ALDH1A1, CD133, CD44—For Survival and Long-Term Follow-Up of Ovarian Cancer Patients

**DOI:** 10.3390/ijms24032400

**Published:** 2023-01-25

**Authors:** Natalia Izycka, Marcin Rucinski, Malgorzata Andrzejewska, Sebastian Szubert, Ewa Nowak-Markwitz, Karolina Sterzynska

**Affiliations:** 1 Department of Gynecology, Obstetrics and Gynecologic Oncology, Division of Gynecologic Oncology, Poznan University of Medical Sciences, Polna 33 St., 60-535 Poznań, Poland; 2 Department of Histology and Embryology, Poznan University of Medical Sciences, Święcickiego 6 St., 61-781 Poznań, Poland

**Keywords:** epithelial ovarian cancer, cancer stem cells, prognosis biomarker, ALH1A1, CD133, CD44

## Abstract

Recurrent disease and treatment-associated chemoresistance are the two main factors accounting for poor clinical outcomes of ovarian cancer (OC) patients. Both can be associated with cancer stem cells (CSCs), which contribute to cancer formation, progression, chemoresistance, and recurrence. Hence, this study investigated whether the expression of known CSC-associated markers ALDH1A, CD44, and CD133 may predict OC patient prognosis. We analyzed their expression in primary epithelial ovarian cancer (EOC) patients using immunohistochemistry and related them to clinicopathological data, including overall survival (OS) and progression-free survival (PFS). Expression of ALDH1A1 was detected in 32%, CD133 in 28%, and CD44 in 33% of cases. While Kaplan–Meier analysis revealed no association of the expression of CD133 and CD44 with PFS and OS, ALDH1A1-positive patients were characterized with both significantly shorter OS (*p* = 0.00022) and PFS (*p* = 0.027). Multivariate analysis demonstrated that the expression of ALDH1A1, FIGO stage III–IV, and residual disease after suboptimal debulking or neoadjuvant chemotherapy correlated with shorter OS. The results of this study identify ALDH1A1 as a potential independent prognostic factor of shorter OS and PFS in EOC patients. Therefore, targeting ALDH1A1-positive cancer cells may be a promising therapeutic strategy to influence the disease course and treatment response.

## 1. Introduction

Epithelial ovarian cancer (EOC) is one of the major causes of cancer-related deaths among women worldwide [1]. Recurrent disease and treatment-related chemoresistance are the two main reasons accounting for poor clinical outcomes of ovarian cancer patients [2]. Occurrence of these factors often results in treatment failure and even death of the patient, manifesting in a significantly shorter progression-free survival (PFS) and overall survival (OS) of EOC patients [3,4,5].

The presence of cancer stem cells (CSCs) in the tumor mass seems to be a key factor contributing to ovarian cancer formation, progression (including metastasis), recurrence, and resistance to different treatment regimens [6,7]. CSCs are a small subpopulation of cancer cells with unique properties, such as self-renewing and differentiating into chemoresistant cancer cells [8,9]. Ovarian CSCs’ potential for unlimited cell division and differentiation into more specialized cell lines and their ability to survive cytotoxic treatment make them essential for tumor survival and recurrence. Moreover, CSC-containing tumors are usually composed of cells characterized by higher resistance to chemotherapy than those found in the primary tumor [10,11]. Thus, targeting ovarian cancer stem cells seems a promising approach that could become the basis of novel EOC therapies. Identifying and targeting CSCs could be crucial to mitigating their potential impact on ovarian cancer treatment success [12]. 

CSCs can be identified by detecting specific surface molecules or intracellular enzyme activity that have previously been associated with similar cell populations in various cancers. Although the phenotype of ovarian CSCs varies, and no definite expression pattern enables their identification, several markers specific to ovarian CSCs have been proposed. Some are cancer-type specific, and some are commonly expressed by all CSCs [13,14]. The most frequently mentioned CSCs markers in EOC include CD44, CD133, CD24, CD117, and ALDH1A [15,16,17].

ALDH1A1 is a protein member of the aldehyde dehydrogenase family. This enzyme participates in cell detoxification, protection against free radicals, and retinol and retinoic acid biosynthesis, regulating stem cell proliferation and differentiation [18,19]. Studies have shown that ALDH1A-positive neoplastic cells exhibit cancer stem properties, such as self-renewal and carcinogenicity. Moreover, their overexpression in various neoplasms, including EOC, has been associated with higher tumor invasiveness, proliferative potential, neoangiogenesis, chemoresistance, and worse survival rates [20,21,22]. Additionally, some in vitro studies have demonstrated that inhibition of ALDH1A1 leads to increased chemosensitivity of cancer cells [23,24]. Hence, identifying ALDH1A independently or in correlation with other CSC markers is an accepted method for detecting these cells in ovarian cancer [20,21,22]. 

CD133 is a human homolog of prominin 1, a mouse transmembrane protein. In human physiology, this protein is expressed on the surface of stem cells, progenitor hematopoietic cells, and cells of various tissues, including the mammary gland, lungs, placenta, and testes [25,26]. In cancer, CD133 is suspected of playing a role in tumorigenesis, progression, and chemoresistance. It is used to identify CSCs in different types of cancer, with its expression usually associated with poor prognosis [27,28,29]. It is one of the most commonly reported CSC markers in ovarian cancer. CD133 expression is usually associated with higher stages of the disease, chemoresistance, and shorter survival time, making it an important marker of OC patient prognosis [28,29]. 

CD44 is a cell surface glycoprotein involved in cellular adhesion, proliferation, and migration. Overexpression of CD44 and its isoforms promotes tumor growth, cell migration, and metastasis, through its participation in the epithelial–mesenchymal transition process (EMT), in a range of neoplasms, including ovarian cancer. Moreover, it suppresses anti-apoptotic activity, leading to the development of cancer chemoresistance [14,30]. In advanced epithelial ovarian cancers, overexpression of CD44 is associated with shorter overall and progression-free survival times due to its promoting effect on both chemoresistance and recurrence of the disease. In turn, low expression of CD44 in tumors is associated with significantly better survival of EOC patients [31,32]. 

A growing body of evidence indicates that some CSC biomarkers may be of diagnostic and prognostic importance in ovarian cancer. However, the results of many studies investigating the role of these proteins in a range of malignancies, including ovarian cancer, are still inconsistent. The actual impact of their expression on the survival of ovarian cancer patients is still being discussed. Therefore, this study aimed to retrospectively investigate the expression of ALDH1A1, CD133, and CD44 in primary epithelial ovarian cancer samples. The results were then compared with clinicopathological data of the patients, including overall survival and long-term follow-up, to evaluate the prognostic and predictive value of these CSC markers.

## 2. Results

### 2.1. Clinicopathological Characteristics

The clinicopathological characteristics of the patients are presented in Table 1. The median patient age was 56 (range 26–84), and 77% of the patients were less than 65 years old. Optimal cytoreduction was achieved in 32 patients (37%), while 54 (63%) patients were only subjected to diagnostic laparoscopy or laparotomy with excisional tumor biopsy and were then scheduled for neoadjuvant chemotherapy. The tumor grade distribution among patients was as follows: 8 (9%) G1, 29 (34%) G2, and 49 (57%) G3. Advanced stage disease was observed in 57 (66%) stage III and 2 (2%) stage IV patients, whereas 22 (26%) and 5 (6%) patients were stages I and II, respectively. Preoperative CA125 serum level lower than 35 U/L was recorded in 7 (8%) patients, and 79 (92%) presented levels higher than 35 U/L. In all, 81% of all patients were classified as platinum-sensitive (no progression during the follow-up or progression occurred six or more months after finishing the first-line chemotherapy).

### 2.2. Immunohistochemical Analysis

**Figure 1:**
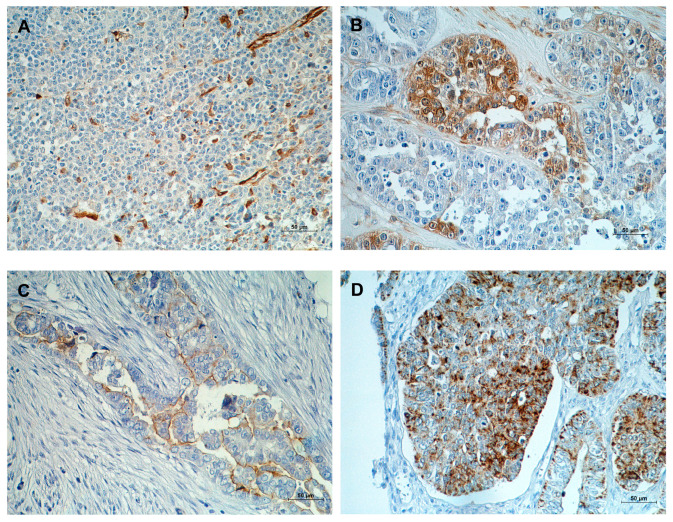

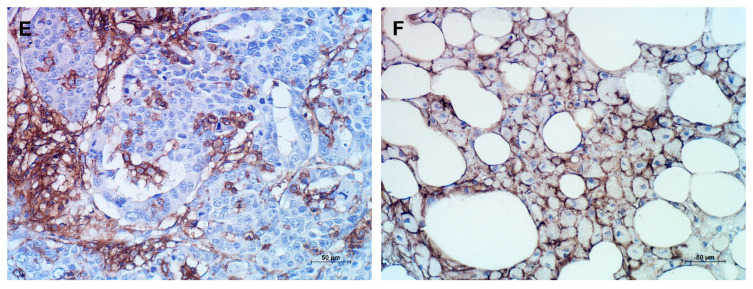
Immunohistochemical expression of (**A**) ALDH1A1—in the cytoplasm of individual cancer cells, (**B**) ALDH1A1—in the cytoplasm of small groups of cancer cells, (**C**) CD133—in the apical cell surface of the tumor cells, (**D**) CD133—in the cytoplasm of the tumor cells, (**E**) CD44—in the cytoplasm and membranes of tumor cells, and (**F**) CD44—in the membranes of tumor cells. The slides were counterstained with hematoxylin. Scale bar = 50 μm.

ALDH1A1 and CD133 positivity was only identified in tumor cells, while CD44 was present in tumor cells and tumor-surrounding stroma cells. The distribution of ALDH1A1 was observed in the cytoplasm, CD44 in the cell membrane, and CD133 in the cytoplasm and/or membrane. The staining patterns for analyzed antigens varied, manifesting as focal, scattered, and diffuse staining (Figure 1). 

According to the assumed evaluation criteria described in the Materials and Methods section, positive ALDH1A1 expression was found in 32% (27/86) of patients. Positive staining for ALDH1A1, in most cases, was visible in individual or small groups of cancer cells (Figure 1A). We could only observe a positive reaction in most tumor cells in a few examined samples (Figure 1B). Expression of ALDH1A1 was mainly seen in the cytoplasm of tumor cells.

Positive CD133 expression was defined in 28% (24/86) of samples. Expression was seen on the apical (endoluminal) cell surface (Figure 1C) and/or in the cytoplasm of solidly arranged tumor cells (Figure 1D).

CD44 expression was detected in 33% (28/86) of patients, both in tumor cells and the surrounding stroma. For the analysis, only tumor cells were taken into consideration. CD44 positivity of tumor cells showed both membranous and cytoplasmic distribution (Figure 1E,F).

The expression of ALDH1A1 was positively correlated with CD133 expression (R Spearman = 0.22, *p* = 0.04). A positive correlation was also observed between CD44 and CD133 expression (R Spearman 0.47, *p* = 4.6^−6^). No significant relation was found between ALDH1A1 and CD44 expression, although the obtained result was close to the assumed statistical significance (R Spearman = 0.21, *p* = 0.055). No significant association was found between ALDH1A1, CD44, and CD133 expression in tumor cells and the examined clinicopathological features, such as age, residual tumor status, grade, FIGO stage, serum CA125, and response to treatment (Table 2) (Fisher’s exact or Chi-square test as appropriate).

### 2.3. Survival Analysis

The median progression-free survival (PFS) for all patients was 28 months (range 0–404), with overall survival (OS) of 46 months (2–136) and with 53 patients (62%) deceased by the end of the study. In all, 32% of patients demonstrated positive ALDH1A1 expression (27/86). The median PFS and OS for this group were 27 (0–85) and 34 months (9–84), respectively. The ALDH1A1-negative group was characterized by PFS of 28 months (0–404) and OS of 58 months (2–136). CD133 expression was recorded in 28% (24/86) of cases, with a median PFS of 18,5 months (3–81) and OS of 41 months (9–103). Patients negative for this antigen had a median PFS of 30 (0–404) and OS of 50 months (2–136). In all, 33% (28/86) of patients were CD44-positive, with a median PFS of 16,5 months (0–85) and a median OS of 40 months (4–103). Median PFS and OS for CD44-negative patients were 30 months (0–404) and 48 months (2–136), respectively.

**Figure 2:**
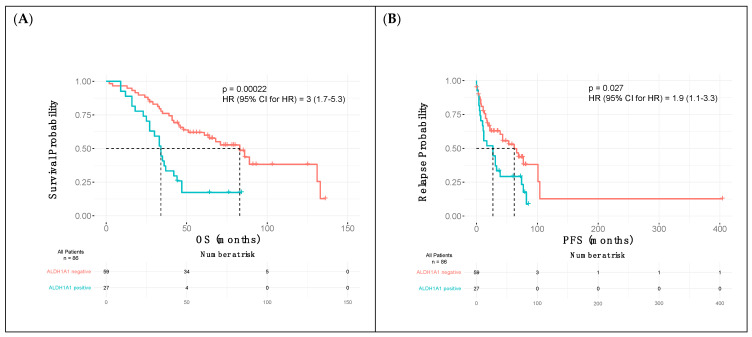
Kaplan–Meier overall survival (**A**) and progression-free survival (**B**) curves according to the ALDH1A1 expression.

PFS and OS curves were estimated based on the status of ALDH1A1, CD133, and CD44 expression, with the difference in patient survival compared using a log-rank test. According to the analysis, the expression of CD133 and CD44 had no statistically significant impact on the overall and progression-free survival in patients with epithelial ovarian cancer. In turn, ALDH1A1-positive patients showed a significantly shorter overall survival time (*p* = 0.00022) and shorter time to relapse (*p* = 0.027) (Figure 2A,B).

The univariate analysis of possible predictors of OS and PFS was conducted using the Cox proportional-hazards regression. The result of this analysis indicated ALDH1A1 expression in tumors as an independent prognostic factor of shorter overall survival and progression-free survival in patients with epithelial ovarian cancer. Moreover, in the multivariate, adjusted survival analysis, we found that the expression of ALDH1A1, FIGO stage III–IV, and suboptimal debulking residual disease following PDS or neoadjuvant chemotherapy were related to shortened overall survival. The results are summarized in Table 3.

## 3. Discussion

Ovarian CSCs are a small subpopulation of cells within tumors, believed to be responsible for cancer growth, spread, development of chemoresistance, and recurrence. Although the role of CSC markers in cancer development is broadly defined, their functionality is still a matter of discussion in the case of ovarian cancer [33]. The existing doubts relate mainly to the role of these markers in disease progression and resistance to chemotherapy. Nonetheless, several studies support using CSCs as a potential targeted ovarian cancer therapy subject [34,35,36]. 

In our research, we studied the prognostic value of the expression of three proteins considered ovarian cancer stem cell markers. We analyzed their expression and related the results to clinical data of EOC patients, including OS and PFS. To our knowledge, this is the first study investigating the prognostic significance of this set of three ovarian CSC markers with clinical presentation of the disease in a cohort of EOC patients, including a long-term follow-up. Our results could be an introduction to further research on the clinical impact of their expression on ovarian cancer patients.

CD133 is one of the most widely described CSC markers in ovarian cancer. However, the results on its prognostic or predictive value still need to be conclusive. Some studies positively link CD133 expression to worse OS and show its relation to some clinicopathological parameters and chemoresistance [37,38]. By contrast, many authors describe no correlation between CD133 expression and patient prognosis [39]. They argue that the expression of this marker is unrelated to other clinicopathological features, such as age, tumor grade, malignant ascites, or CA125 serum level [40]. Our study follows the latter, as no significant association was found between CD133 expression in tumor cells and clinicopathological features (age, residual tumor status, grade, FIGO stage, serum CA125, and response to treatment). Statistical analysis also revealed that the tumor expression of this marker shows no significant correlation with OS and PFS, which remains in line with the results of others [39,40]. Due to the controversial results on the role of CD133 in ovarian cancer, additional studies are needed to shed more light on its usefulness in predicting patient survival and chemosensitivity.

Literature data on the role of CD44 in the progression of ovarian cancer, and its relation to clinical data, are also mixed and often contradictory. Based on the literature, it is difficult to unequivocally recognize its expression as a prognostic indicator for ovarian cancer patients [41,42]. Nonetheless, some researchers advocated the expression of CD44 as a biomarker associated with some clinicopathological features of the tumor, such as high-grade, more advanced FIGO stage, or the presence of residual disease and drug resistance [43,44]. Moreover, according to several authors, increased expression of CD44 was associated with significantly worse OS and PFS of OC patients [45]. By contrast, other studies demonstrated that high expression of CD44 correlated with better OC patient outcomes. Furthermore, Bartakova et al. concluded that CD44 was a prognostic factor for neither OS nor PFS [46,47,48]. Our results show no correlation between CD 44 expression and clinicopathological features such as age, residual tumor status, grade, FIGO stage, serum CA125, and response to treatment. The contradictory research results may result from methodological differences, where researchers use different antibodies and detection methods. Therefore, the results obtained for CD44 expression did not contradict nor clarify the potential prognostic value of this biomarker in ovarian cancer patients. Further investigation of a larger group of EOC patients is required to define its prognostic or predictive significance.

Differently from CD133 and CD44, we identified that ALDH1A1 expression was significantly associated with patients’ outcomes. Again, literature data present conflicting results on the impact of ALDH1A1 expression and the prognosis of EOC patients. On one hand, some studies demonstrate a correlation between ALDH1A1 expression and unfavorable prognosis [21,49,50]. According to other sources, the expression of ALDH1A1 relates to a favorable prognosis [51], less advanced FIGO stage, better tumor differentiation, and better survival rates [2]. The results of our research are consistent with those of Tao et al., who reported that the expression of ALDH1A1 was associated with shorter OS and PFS of OC patients [38]. Our immunohistochemical results support the prognostic significance of ALDH1A1 expression due to its negative impact on overall survival. As PFS was also significantly shorter in the group of ALDH1A1-positive patients, using this CSC biomarker to predict chemotherapy response in clinical practice might be valuable. As already proven in our previous research and by others, the ALDH1A1-positive populations of ovarian cancer cells were more resistant to chemotherapeutic drugs used in the standard treatment of ovarian cancer patients [6,21,23,24,52,53]. Hence, the presence of ALDH1A1-positive cells may influence the disease course and its response to treatment. Additionally, our multivariate analysis revealed that the expression of ALDH1A1, FIGO stage III–IV, and suboptimal debulking-related residual disease following PDS or neoadjuvant chemotherapy were all risk factors for shortened overall survival. Based on these results, ALDH1A1 expression in tumors should be considered an independent prognostic factor of shorter overall survival and progression-free survival in patients with epithelial ovarian cancer. Nevertheless, the association between ALDH1A1 expression and other clinical features of ovarian cancer, including grading, FIGO stage, or residual tumor status, has not been proven in our study. ALDH1A1 is considered a universal cancer stem cell marker and already serves as a therapeutic target for different selective inhibitors due to its role in chemoresistance [54,55,56]. Therefore, ADLH1A1-targeted therapy has significant potential to play a role in overcoming chemotherapy resistance and improving patient outcomes.

## 4. Materials and Methods

### 4.1. Patient Characteristics

The study included 86 patients diagnosed with epithelial ovarian cancer, treated between 2010 and 2018 in the Gynecologic Oncology Department, Poznan University of Medical Sciences. All the samples were obtained during primary cytoreductive surgery (PDS) or diagnostic laparoscopy/laparotomy. The study included a retrospective immunohistochemical examination of archival samples, followed by the analysis of the clinicopathological features of patients, including age, diagnosis, FIGO stage, tumor grade, residual tumor status, treatment protocols, preoperative cancer antigen 125 (CA125) serum levels, response to treatment, time of relapse, and time of death or last follow-up. 

All patients underwent primary surgery to achieve optimal cytoreduction (residual disease less than 1 cm). Following the surgical procedure with optimal debulking, patients received adjuvant chemotherapy composed of paclitaxel and carboplatin. If optimal debulking was not possible, a tumor sample was obtained for histopathological evaluation, and neoadjuvant chemotherapy composed of paclitaxel and carboplatin with or without bevacizumab was introduced, according to the ovarian cancer treatment guidelines (NCCN Guidelines for Ovarian Cancer V.1.2022) [57,58,59].

If a satisfactory response to neoadjuvant chemotherapy was observed, patients underwent interval debulking surgery. This study was approved by the Bioethics Committee at the Poznan University of Medical Sciences.

### 4.2. Immunohistochemical Analysis

Immunohistochemical analysis of ALDH1A1, CD133, and CD44 expression was performed on formalin-fixed, paraffin-embedded human ovarian cancer samples. Briefly, 5 µm sample sections were deparaffinized in xylene, hydrated in decreasing alcohol concentrations, and washed. CD133 antigen retrieval was performed by heating the slides in 10 mM citrate buffer for 10 min. Next, for all the examined antigens, endogenous peroxidase activity was blocked through 30 min submersion in 3% H_2_O_2_. The slides were then incubated with normal goat serum (Dako, Glostrup, Denmark) for 30 min and incubated overnight at 4 °C with the primary antibodies anti-ALDH1A1 (mouse monoclonal, H4 clone, 1:200 dilution, Santa Cruz Biotechnology, Santa Cruz, CA, USA), anti-CD44 (mouse monoclonal, DF1485 clone, 1:100 dilution, Dako, Glostrup, Denmark), and anti-CD133 (rabbit polyclonal, 1:200 dilution, Abcam, Cambridge, UK). Primary antibodies were detected using the EnVision Detection System (Dako REALTMEnVisionTM Detection System peroxidase/DAB+, Rabbit/Mouse, Dako, Glostrup, Denmark) for 30 min at room temperature. Finally, the slides were rinsed with distilled water, counterstained with hematoxylin, dehydrated in increasing alcohol concentrations, and mounted. Immunohistochemical staining was analyzed under an Olympus BH-2 optical microscope, coupled with a digital camera (LUCIA Image 5.0 software, Nikon, Tokyo, Japan), and assessed by two independent investigators. Positive cells were scored in ten non-overlapping microscopic fields at a magnification of 400×.

Two independent investigators performed immunohistochemical analysis, blinded to the clinical data, and according to the following criteria. The expression of CD133 and CD44 was assessed, focusing on the percentage of positive tumor cells and staining intensity. The immunoreactivity was interpreted as 0 (no or less than 10% cells positive), 1+ (>10% cells with minimal staining), 2+ (>10% cells with moderate staining), and 3+ (>10% cells with strong staining). ALDH1A1 cases were defined as positive when more than 1% of cancer cells demonstrated positive expression. The 1% cut-off value was established based on the literature data, which assumes that even a small number of ALDH1A1-positive cells may be responsible for the stem cell-like tumor features. For the statistical analyses, CSCs’ marker expression scores were dichotomized into two groups: 0 (no expression) and 1 (1+, 2+, or 3+ immunoreactivity score).

### 4.3. Statistical Analysis

The distribution of examined cases according to clinicopathological features was performed using Fisher’s exact or Chi-square test as appropriate.

Survival analyses were conducted using the Kaplan–Meier survival curves, with the difference in patient survival compared using a log-rank test. Univariate analysis of possible OS and PFS predictors was performed using the Cox proportional hazard regression. Multivariate survival analysis was also conducted using the Cox proportional-hazards regression, with the entry method of variable entry, to evaluate the prognostic significance of CSC markers (ALDH1A1, CD133, CD44) expression on patient survival. We investigated the impact of the following factors on patient survival: FIGO stage of the disease (I–II vs. III–IV), tumor grade, residual disease following PDS (optimal cytoreduction vs. no surgery or suboptimal debulking), patient age (below and above 65), and CA125 levels (below and above 35 IU/mL). All the factors mentioned above accounted for model design. According to the stepwise method, significant variables were entered into the model sequentially. After entering, the variable was rechecked, with nonsignificant variables removed. All statistical values with a *p*-value lower than 0.05 were considered statistically significant.

Overall survival was calculated from the date of treatment initiation to the date of death or last follow-up. Progression-free survival was considered the time from the date of last first-line chemotherapy admission to the date of progression or last follow-up. Response to treatment was classified into two categories: platinum-sensitive—no progression was recorded during the follow-up or progression occurred six or more months after finishing the first-line chemotherapy; and platinum-resistant—when progression was reported within six months after discontinuing the treatment.

The statistical analysis was performed using the R programming language, with the “ggplot2” library used for graph design (ver. 4.2.1; R Core Team 2022, Vienna, Austria), MedCalc 11.4.2.0. (MedCalc Software, Seoul, Republic of Korea), and GraphPad InStat 3.06 (GraphPad Software Inc., San Diego, CA, USA).

## 5. Conclusions

The results of this study identify ALDH1A1 as an independent prognostic factor of shorter OS and PFS in EOC patients. Targeting ALDH1A1-positive cancer cells may be a promising therapeutic strategy, influencing disease course and response to treatment. A clinical value of CD133 and CD44 expression in predicting disease progression was not confirmed, which suggests that further investigation on a larger group of patients is required to clarify their prognostic significance.

## Figures and Tables

**Table 1:** Clinicopathological characteristics of patients.

Characteristics	Frequency
Age	
<65	66 (77%)
≥65	20 (23%)
Residual tumor	
<1	32 (37%)
≥1	54 (63%)
Grade	
G1	8 (9%)
G2	29 (34%)
G3	49 (57%)
FIGO	
I	22 (26%)
II	5 (6%)
III	57 (66%)
IV	2 (2%)
FIGO I–II	26 (30%)
FIGO III–IV	60 (70%)
Histological type	
serous	69 (80%)
endometrioid	7 (8%)
mucinous	5 (6%)
clear cell	5 (6%)
CA125 preoperative serum level	
<35 U/L	7 (8%)
≥35 U/L	79 (92%)
PFS	
<6	15 (17%)
6–12	11 (13%)
>12	60 (70%)
Response to treatment	
platinum-resistant	16 (19%)
platinum-sensitive	70 (81%)

**Table 2:** Analysis of the relationship between ALDH1A1, CD44, and CD133 expression and clinicopathological characteristics.

Variable	N	ALDH1A1 Negative	ALDH1A1 Positive	*p* Value	CD44 Negative	CD44 Positive	*p* Value	CD133 Negative	CD133 Positive	*p* Value
Age < 65	66	47 (71%)	19 (29%)	0.41	43 (65%)	23 (35%)	0.590	46 (70%)	20 (30%)	0.570
Age ≥ 65	20	12 (60%)	8 (40%)		15 (75%)	5 (25%)		16 (80%)	4 (20%)	
Residual tumor < 1	32	22 (69%)	10 (31%)	1.00	25 (78%)	7 (22%)	0.150	28 (88%)	4 (12%)	0.024
Residual tumor ≥ 1	54	37 (69%)	17 (31%)		33 (61%)	21 (39%)		34 (63%)	20 (37%)	
G1	8	5 (62%)	3 (38%)	0.93	7 (88%)	1 (12%)	0.062	8 (100%)	0 (0%)	0.110
G2	29	20 (69%)	9 (31%)		23 (79%)	6 (21%)		22 (76%)	7 (24%)	
G3	49	34 (69%)	15 (31%)		28 (57%)	21 (43%)		32 (65%)	17 (35%)	
FIGO I–II	26	17 (65%)	9 (35%)	0.80	20 (77%)	6 (23%)	0.320	22 (85%)	4 (15%)	0.120
FIGO III–IV	60	42 (70%)	18 (30%)		38 (63%)	22 (37%)		40 (67%)	20 (33%)	
Ca125 < 35 U/L	7	5 (71%)	2 (29%)	1.00	5 (71%)	2 (29%)	1.000	6 (86%)	1 (14%)	0.670
Ca125 ≥ 35 U/L	79	54 (68%)	25 (32%)		53 (67%)	26 (33%)		56 (71%)	23 (29%)	
PFS < 6	15	9 (60%)	6 (40%)	0.64	7 (47%)	8 (53%)	0.120	11 (73%)	4 (27%)	1.000
PFS 6–12	11	7 (64%)	4 (36%)		7 (64%)	4 (36%)		8 (73%)	3 (27%)	
PFS ≥ 12	60	43 (72%)	17 (28%)		44 (73%)	16 (27%)		43 (72%)	17 (28%)	
Platinum-resistant	16	9 (56%)	7 (44%)	0.25	8 (50%)	8 (50%)	0.140	12 (75%)	4 (25%)	1.000
Platinum-sensitive	70	50 (71%)	20 (29%)		50 (71%)	20 (29%)		50 (71%)	20 (29%)	

**Table 3:** Multivariate Cox regression analysis using overall survival as a dependent variable in ovarian cancer patients.

Overall Survival (OS)		
	HR (95% CI for HR)	*p* Value
ALDH1A1 (positive)	3.1 (1.6–6)	0.0007 *
CD44 (positive)	1 (0.5–2.1)	0.9852
CD133 (positive)	0.7 (0.3–1.4)	0.2952
FIGO III–IV	2.6 (1–6.5)	0.0419 *
Grade	1.7 (1–2.8)	0.0628
Suboptimal debulking or neoadjuvant chemotherapy	2.7 (1.2–6.1)	0.0206 *

* Indicates statistical significance, *p* < 0.05.

## Data Availability

Data presented in this study are available on request from the corresponding author.
